# Differentiating brucella spondylitis from tuberculous spondylitis by the conventional MRI and MR T2 mapping: a prospective study

**DOI:** 10.1186/s40001-021-00598-4

**Published:** 2021-10-28

**Authors:** Hui Guo, Siqin Lan, Yuanlin He, Maijudan Tiheiran, Wenya Liu

**Affiliations:** Medical Imaging Center, Xinjiang Medical University Affiliated First Hospital, Urumqi, 830054 People’s Republic of China

**Keywords:** Brucellosis, Tuberculous, Spondylitis, T2 mapping, Quantitative

## Abstract

**Background:**

Brucella spondylitis (BS) and tuberculous spondylitis (TS), caused initially by bacteremia, are the two leading types of granulomatous spinal infections. BS is easy to miss or may be misdiagnosed as TS. Our purpose aims to differentiate BS from TS in conventional MR imaging and MR T2 mapping.

**Methods:**

We performed on 26 BS and 27 TS patients conventional MR imaging and MR T2 mapping. We analyzed the features in conventional MR imaging and measured T2 values of the lesion vertebrae (LV) and unaffected adjacent vertebrae (UAV) in BS and TS patients, respectively.

**Results:**

There were no significant differences in sex, age, national between BS and TS. There was significantly lower severity of vertebral destruction, vertebral posterior convex deformity, dead bone, and abscess scope in BS when compared to TS (*p*  <  0.001, *p*  =  0.048, *p*  <  0.001, *p*  <  0.001, respectively). The vertebral hyperplasia was significantly higher in BS when compared to TS (*p*  <  0.001). The T2 value of the LV with BS was markedly higher than that in the UAV with BS and that in the LV and UAV with TS (*p*  <  0.001, *p*  <  0.037, *p*  <  0.001, respectively). The T2 value of the LV with TS was significantly higher than that of the UAV in TS and BS (*p*  <  0.001, *p*  <  0.001, respectively). There were no significant differences in the T2 value of the UAV between BS and TS (*p*  =  0.568).

**Conclusions:**

The qualitative and quantitative evaluation may differentiate BS from TS. The conventional MR imaging helps to distinguish BS from TS by several distinctive features. MR T2 mapping has the additional potential to provide quantitative information between BS and TS.

## Key points

Conventional MR imaging has several distinctive features to distinguish BS from TS.

MR T2 mapping has the additional potential to provide quantitative information between BS and TS.

MR T2 mapping might be a valuable tool for a non-invasive and quantitative technique.

## Background

Brucella spondylitis (BS) and tuberculous spondylitis (TS), caused initially by bacteremia, are the two leading types of granulomatous spinal infections [1]. They have some common clinical manifestations, including back pain, fever, and increased inflammatory markers. It is challenging to precisely distinguish clinically between the two groups [2]. BS is easy to misdiagnose as TS. Conventional MRI can detect changes in the signals and morphology in the vertebrae, which are usually qualitative. However, MR T2 mapping can help visualize and quantitatively access the water content of vertebral bodies [3]. T2 mapping has been used to evaluate lumbar intervertebral disc degeneration [4–8] and only studied vertebra injury in spinal tuberculosis [9]. The study aimed to explore whether qualitatively–quantitatively differentiate BS from TS on conventional MRI and MR T2 mapping.

## Materials and methods

### Study population

**Fig. 1:**
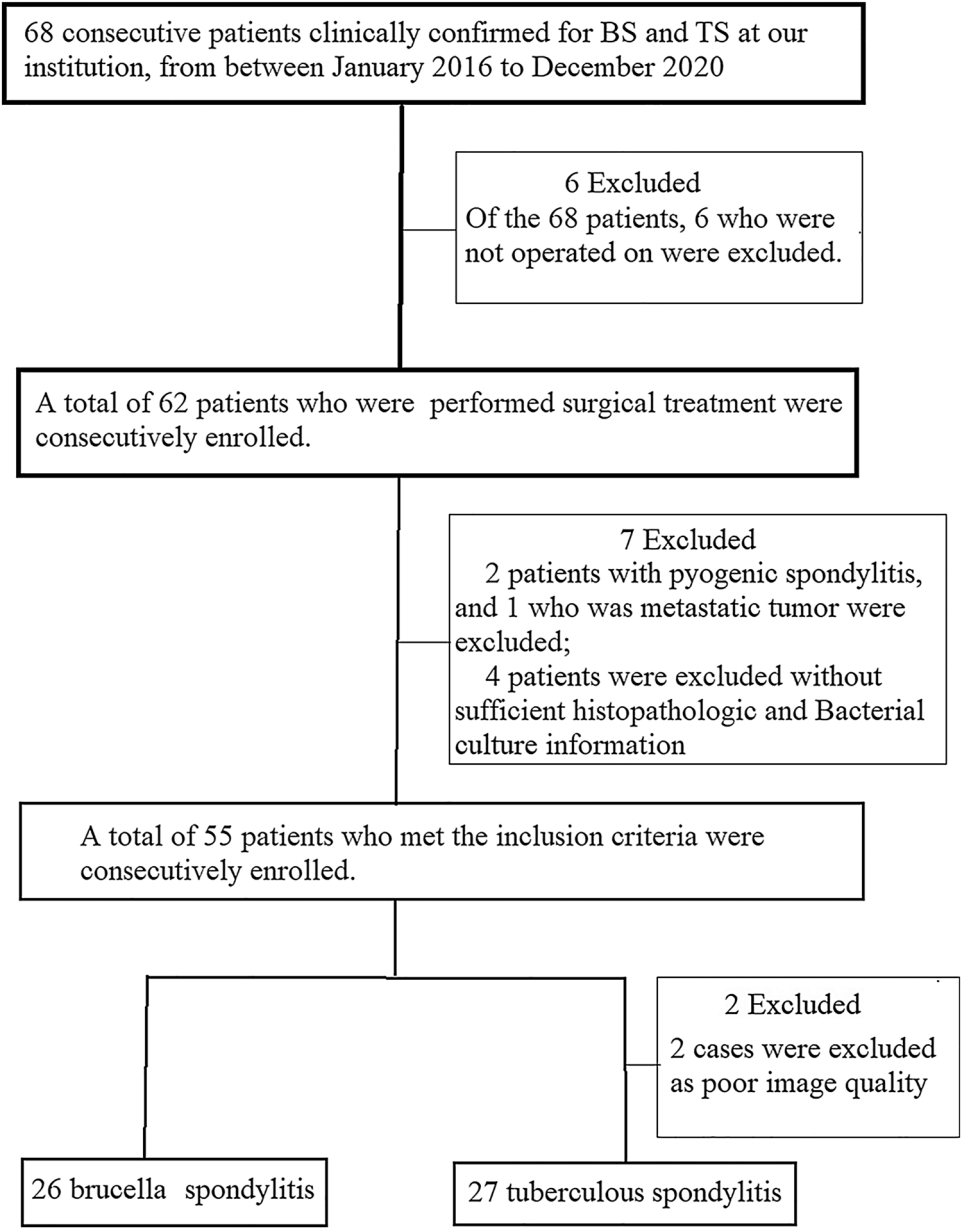
Flowchart of the study population with brucella spondylitis and tuberculous spondylitis

This is a prospective clinical study. Patients who were clinically confirmed for BS and TS between January 2018 and December 2020 were initially considered eligible for our research (*n*  =  68). All participants provided written informed consent. Ethical approval for the study was obtained from the ethical review committee for our hospital. Inclusion criteria were as follows [10–12]: (a) diagnosis of TS was confirmed by biopsy on basing caseation granulomatosis on histopathological examination or the presence of acid-fast bacilli or the tuberculosis bacilli growth in cultures; (b) diagnosis of BS was based on the Brucella agglutination titer test (≥  1:160) and isolation of Brucella species from blood, bone marrow, or tissues; (c) all patients were operated with sufficient histopathologic and bacterial culture information. A total of 55 patients who met the inclusion criteria were consecutively enrolled. 2 patients were excluded as poor image quality. Finally, we included 53 patients who were performed in our study, among whom BS patients (*n*  =  26) and TS patients (*n*  =  27) (Fig. 1).

### MRI protocol

Conventional magnetic resonance imaging (MRI) and MR T2 mapping sequences were carried on in all patients and whole spine MRI studies were performed. MRI scans were performed using a 1.5-T MR scanner (Siemens Healthcare, Erlangen, Germany). The parameters for conventional MRI and MR T2 mapping sequences are shown in Table 1.

**Table 1:** Conventional MRI and MR T2 mapping parameters of all patients

Sequence	TR (ms)	TE (ms)	Layer chick (mm)	Layer distance (mm)	Layer number	FOV (mm)	Matrix
T1WI	600	9.5	4.0	1	13	320	256 × 256
T2WI	3000	88	4.0	1	13	320	256 × 256
FST2WI	3600	83	4.0	1	13	320	256 × 256
T2 mapping	2400	26	3.0	1	17	260	256 × 256

### Image analysis

MRI finding included the level of involvement, number of the affected vertebra, MRI signal (hypointense signal on T1WI, hyperintense signal on T2WI, and hyperintense signal on STIR), vertebral change (destruction, wedge, hyperplasia, bead bone, posterior convex deformity), intervertebral space, and abscess (paravertebral abscess, epidural abscess, psoas abscess, abscess scope), vertebral appendage lesion. Vertebral destruction was defined as a vertebral structure loss of worm-etched or patchy. Vertebral wedge was defined as the front edge of the vertebra is narrower than the back edge, and the vertebra was flattened. The spinal posterior convex deformity was defined as severe vertebral damage, with significant vertebral wedge changes, resulting in considerable kyphosis of the spine. Vertebral appendage lesion was defined as bone edema or bone destruction of the appendage. Bead bone was defined as necrotic bone. Vertebral hyperplasia was defined as the appearance of a spur or osteophyte. MR images were analyzed by an attending physician and an associate chief physician, and the consistency of image evaluation was evaluated, being blinded to histopathological results.

Using the Function Tool 2 software on the post-processing workstation, we selected a region of interest (ROI) with an area of 60 mm^2^ on the T2 mapping image and generated T2 values automatically. The ROI was placed in the middle three layers, where the lesion showed the best. Then, we obtained the T2 average value three times, which was measured repeatedly for the lesion vertebra (LV) and the unaffected adjacent vertebra (UAV) with BS and TS patients.

### Statistical analysis

The information of sex, national, and MRI finding was expressed as the percentages and frequencies. In addition, we collected the information of age and measured T2 values of LV and UAV in the BS and TS patients. And these data concerning age and T2 values of LV and UAV were expressed as mean values and standard deviations. The Chi-square analyzed the differences between the two groups, and the Student’s *t *test analyzed all mean value of the differences between the two groups. A *p *value of less than 0.05 indicated a significant difference. The Kappa coefficient was calculated by two physicians using a consistency test.

## Results

The demographic characteristics in the two groups are shown in Table 2. There were no significant differences in sex, age, national between the BS and the TS.

**Table 2:** The demographic characteristics in BS and TS patients

No. of patients	BS	TS	*p* value
Sex			0.132
Male	15 (57.69%)	10 (37.04%)	
Female	11 (42.31%)	17 (62.96%)	
Age			
Mean ± SD	50.95 ± 13.41	47.33 ± 15.43	0.682
Range of age	31–72	18–69	
National			0.451
Uygur	13 (66.67%)	18 (66.67%)	
Han	8 (22.22%)	5 (18.52%)	
Other	5 (11.11%)	4 (14.81%)	

*BS* brucellosis spondylitis; *TS* tuberculous spondylitis

The image quality of the two physicians was consistent, and the Kappa coefficient was 0.875. MRI findings in the two groups are shown in Table 3. There were significant differences in the site of involvement, vertebral destruction, vertebral posterior convex deformity, dead bone, vertebral hyperplasia, intervertebral space change, and abscess findings between BS and TS (*p*  <  0.05). It was significantly lower in the severe vertebral destruction, vertebral posterior convex deformity, dead bone, and abscess beyond the vertebra lesion with BS when compared to TS (*p*  <  0.001, *p*  =  0.005, *p*  =  0.048, *p*  <  0.001, *p*  <  0.001, respectively). The vertebral hyperplasia was significantly higher in BS when compared to TS (*p*  <  0.001). The lumbar vertebras (69.23%) were the most common in BS. The thoracolumbar vertebras (33.33%) and lumbar vertebras (33.33%) were the most common in TS. It was significantly higher in the normal intervertebral space with BS (42.31%) when compared to TS (7.41%) (*p*  <  0.05), and the narrow intervertebral space was distinctly lower with BS (57.69%) when compared to TS (81.48%) (*p*  <  0.05). The paravertebral abscess was higher with BS (65.38%) when compared to TS (22.22%) (*p*  <  0.05), and it was markedly lower in the psoas abscess with BS (0.00%) when compared to TS (66.67%) (*p*  <  0.05). There were no significant differences in the number of the affected vertebra, MRI signal, vertebral wedge, vertebral appendage lesion between BS and TS (*p*  >  0.05).

**Table 3:** Comparison of MRI findings between brucella spondylitis and tuberculous spondylitis

MRI findings	BS [n (%)]	TS [n (%)]	*χ*^2^ value	*p* value
Site of involvement			11.106	0.025
Cervical spine	0	1 (3.70%)		
Thoracic spine	2 (14.18%)	6 (22.22%)		
Thoracolumbar spine	2 (14.18%)	9 (33.33%)		
Lumbar spine	18 (69.23%)	9 (33.33%)		
Lumbosacral spine	4 (15.38%)	2 (7.40%)		
Number of affected vertebra			0.670	0.715
1	1 (3.85%)	1 (3.70%)		
2	23 (88.46%)	22 (81.48%)		
≥ 3	2 (7.69%)	4 (14.81%)		
MRI signal				
Hypointense signal on T1WI	23 (88.46%)	26 (96.29%)	1.165	0.280
Hyperintense signal on T2WI	14 (53.85%)	10 (37.04%)	1.510	0.219
Hyperintense signal on STIR	18 (69.23%)	21 (77.78%)	0.498	0.480
Vertebral change				
Vertebral destruction			20.974	< 0.001
Mild (≤ 1/3)	23 (88.46%)	8 (29.63%)		
Severe (> 1/3)	2 (7.41%)	19 (70.37%)		
Vertebral wedge			0.229	0.632
≤ 1/2	7 (26.92%)	20 (74.07%)		
> 1/2	1 (3.85%)	5 (18.52%)		
Posterior convex deformity	1 (3.85%)	6 (22.22%)	3.902	0.048
Vertebral appendage lesion	3 (11.54%)	5 (18.52%)	0.504	0.478
Dead bone	0 (0.00%)	13 (48.15%)	16.587	< 0.001
Vertebral hyperplasia	25 (96.15%)	8 (29.63%)	24.948	< 0.001
Intervertebral space			10.540	0.005
Normal	11 (42.31%)	2 (7.41%)		
Narrow	15 (57.69%)	22 (81.48%)		
Disappear	0 (0.00%)	3 (11.11%)		
Abscess			22.945	< 0.001
Paravertebral abscess	17 (65.38%)	6 (22.22%)		
Epidural abscess	9 (34.62%)	8 (29.62%)		
Psoas abscess	0 (0.00%)	18 (66.67%)		
Abscess scope			27.451	< 0.001
Beyond the vertebra lesion	1 (5.88%)	17 (94.44%)		
In the vertebra lesion	16 (94.12%)	1 (5.56%)		

*BS* brucella spondylitis; *TS* tuberculous spondylitis; *STIR* short-tau inversion recovery

**Fig. 2:**
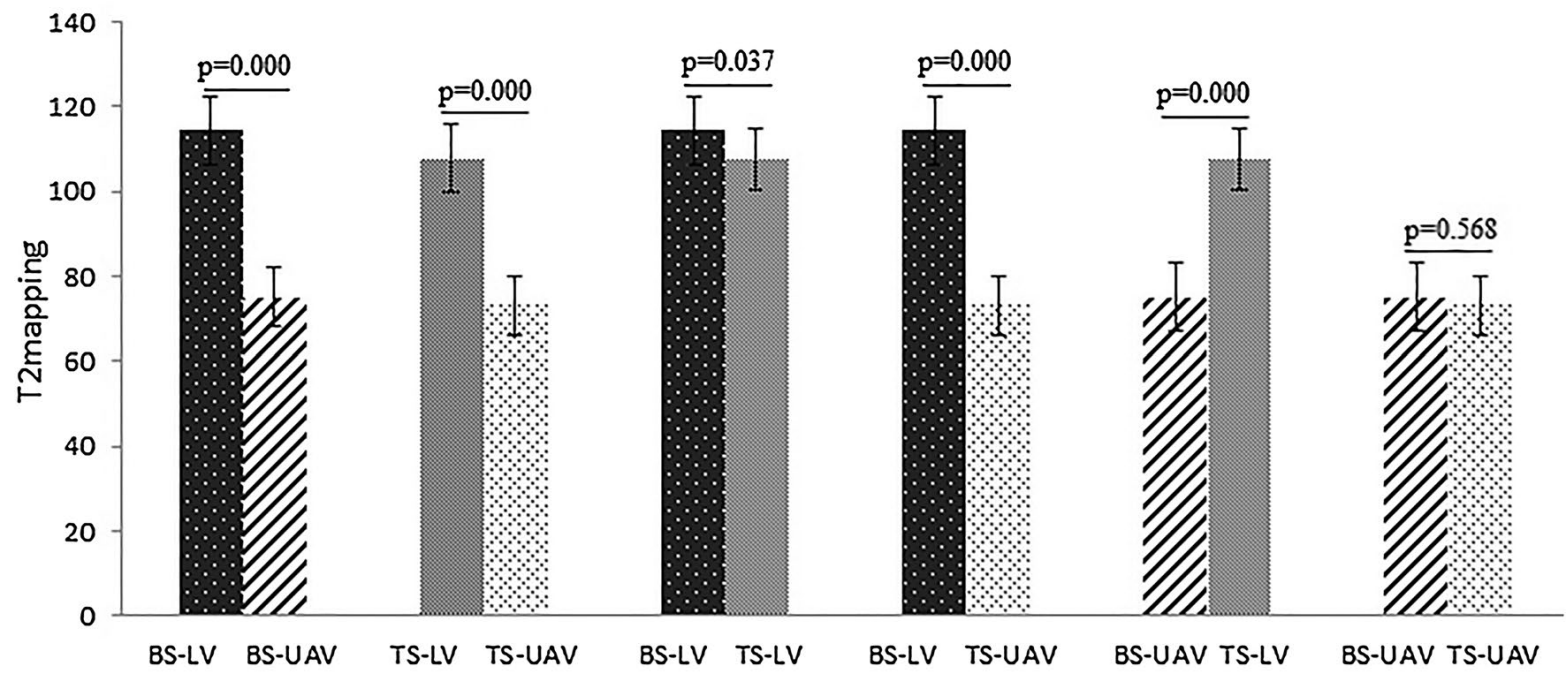
This shows group *t* test results of T2 values for the lesion vertebra and the unaffected adjacent vertebra between brucella spondylitis and tuberculous spondylitis

**Fig. 3:**
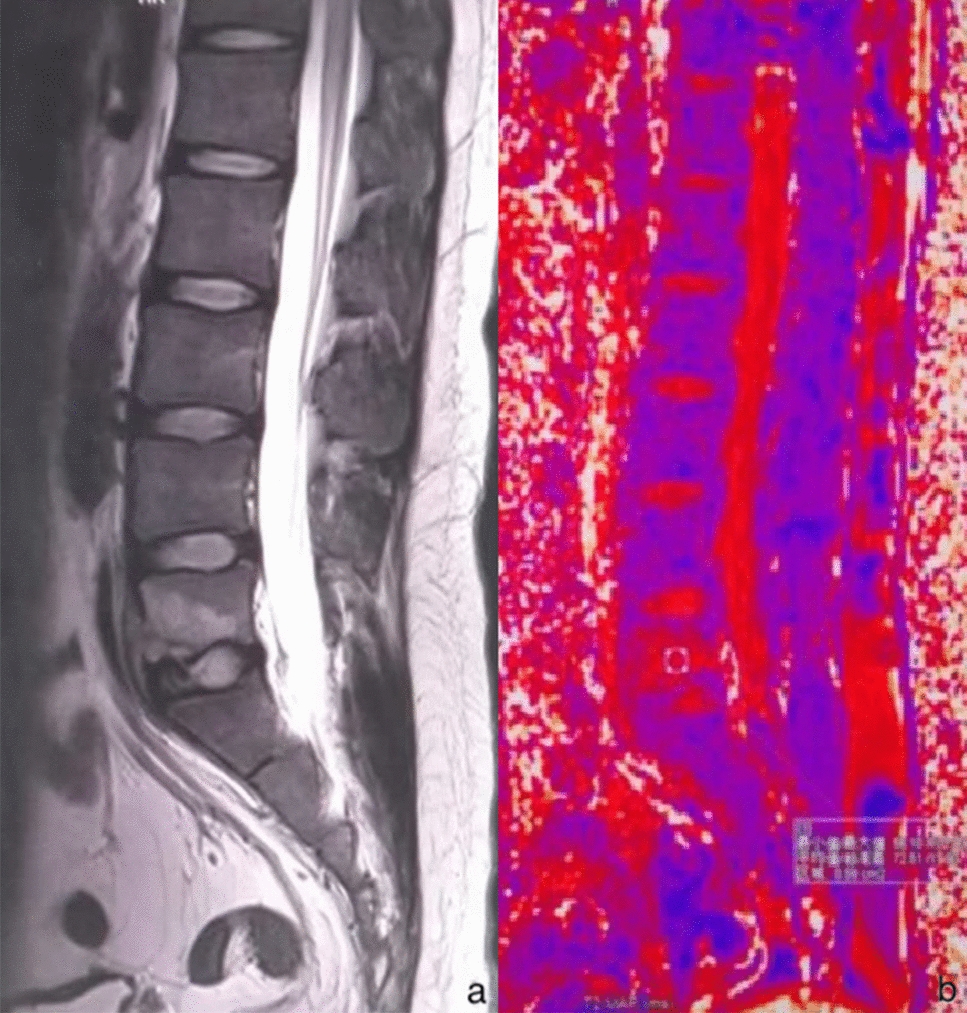
Sagittal MR T2WI (**a**) showed a high signal of the fifth lumbar vertebra, and sagittal MR T2 mapping (**b**) showed the measurement of the fifth lumbar vertebral lesion

The T2 values of the LV and UAV with BS and TS are shown in Fig. 2. The T2 value of the LV with BS was markedly higher than those in the LV with BS and those in the LV and UAV with TS (*p*  <  0.001, *p*  <  0.037, *p*  <  0.001, respectively). The T2 value of the LV with TS was significantly higher than those of the UAV with TS and BS (*p*  <  0.001, *p*  <  0.001, respectively). There were no significant differences in T2 values of the UAV between BS and TS (*p*  =  0.568). Figure 3 presents the measurement of a lumbar vertebral lesion.

## Discussion

This study demonstrated that the qualitative and the quantitative evaluation might differentiate BS from TS. Several distinctive features (site of involvement, vertebral destruction, posterior convex deformity, bead bone, vertebral hyperplasia, intervertebral space change, and location of abscess) were identified. They can distinguish BS from TS in conventional MR imaging. The T2 value of the LV with BS was markedly higher than that in the LV with TS by using the T2 mapping technique.

BS and TS are still considered public health problems worldwide, particularly in developing countries [11, 12]. In this study, there were no significant differences in sex, age, national between BS and TS. The difference in age was inconsistent with this reported in Liu’s study [13]. The reason may be related to the sample size.

**Fig. 4:**
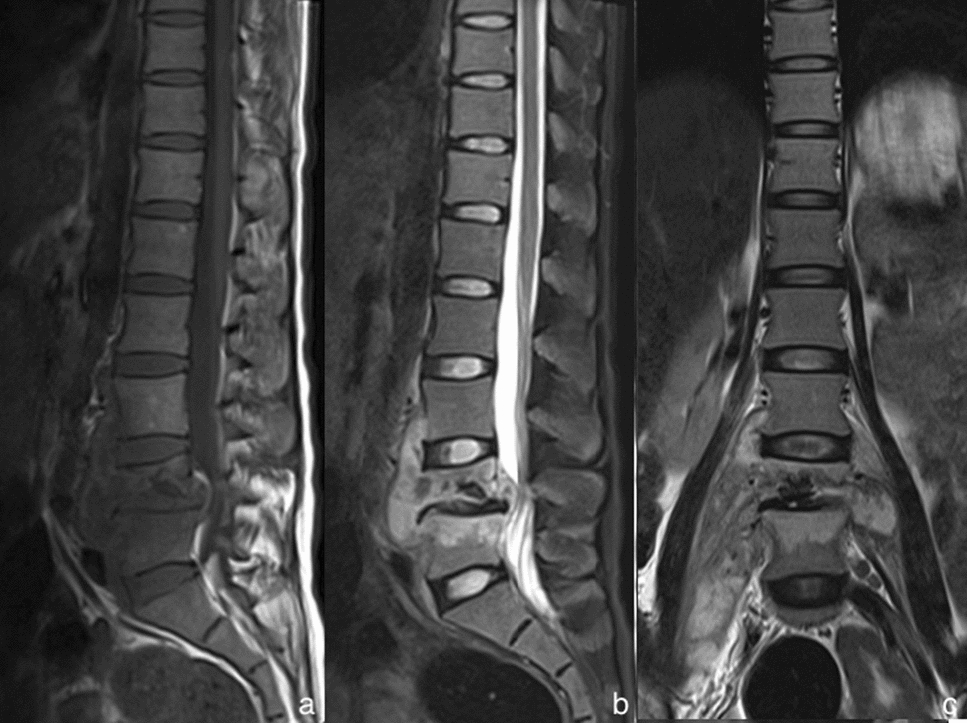
The MRI of a 23-year-old woman complaining of continuous lumbago with tuberculous spondylitis. The sagittal MRI T1WI (**a**), the sagittal MRI with fat saturation (**b**) and the coronal MRI T2WI (**c**) presented a combination of MRI findings: the lesions, in lumbar vertebral bodies 4 and 5, showed hypointense signal in T1WI and hyperintense signal in T2WI. They revealed typical vertebral bodies destruction and collapse, along with the vertebral posterior convex deformity and a narrowed intervertebral space. There also existed extensive psoas abscess and paraspinal abscess around

**Fig. 5:**
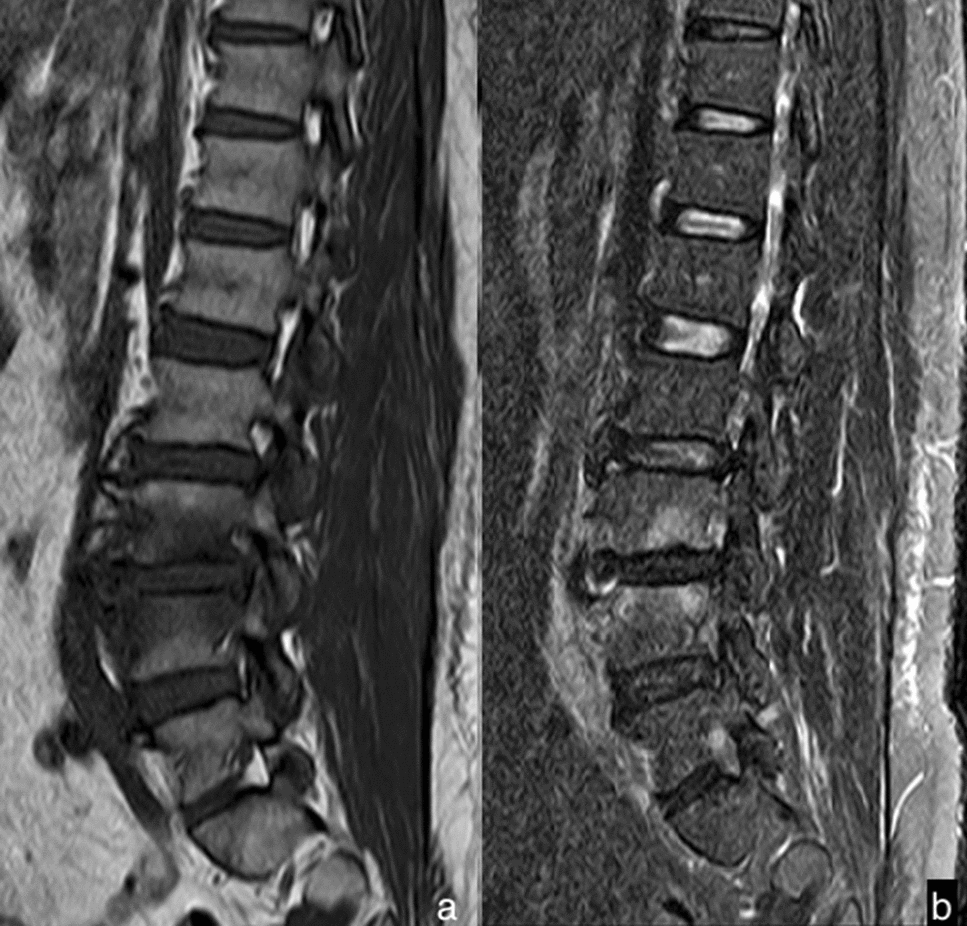
The MRI of a 47-year-old man suffering from weakness with brucella spondylitis. The lesions showed hypointense signal in T1WI (**a**) and slightly hyperintense signal in sagittal MRI with fat saturation (**b**) in lumbar vertebral bodies 4 and 5. Distinct vertebral hyperplasia and small abscesses could be found

The early diagnosis and effective cure become critically important to minimize spinal deformity and permanent neurologic deficiencies. Due to the similarities in the clinical signs and laboratory data, a proportion of patients may be misdiagnosed [14], it is challenging to distinguish BS from TS. In the current study, 69.23% of patients with BS were located in the lumbar, consistent with previous studies [15–17]. However, the majority of TS cases (55.55%) were located in the lower thoracic region, and it was consistent with those in Turunc et al. [2] and Jung et al. [18]. Through the analysis of vertebra and intervertebral space, it was significantly lower in the severe vertebral destruction, vertebral posterior convex deformity, dead bone, and narrow—disappear change of intervertebral space in the patients with BS (7.41%, 3.85%, 0.00%, 57.69%, respectively) than those in TS (70.37%, 22.22%, 48.15%, 92.59%, respectively). This widespread destruction in TS may result from the rapid involvement of the endplate (inflammatory reaction). With the progress of TS, the vertebras were destroyed increasingly severely. The wedge changes of the involved adjacent vertebras resulted in the vertebral posterior convex deformity, along with a narrow or disappeared change in the intervertebral space (Fig. 4a–c). Our study found that the vertebral destruction was significantly severer in TS when compared to BS. The findings were consistent with those in Yang et al. [19] and Liu et al. [13]. A pathologic study pointed out that there was proteinase activity to destroy the disc and vertebra in TS. The vertebral erosion in TS was caseating granulomas and dead bone without new bone formation [20]. As a result, the vertebra in TS presented a severe collapse on MR images, however, it is rare in BS. Similar findings had also been reported by Tali et al. [21]. BS is more common in the mild and focal vertebral destruction, which is agreement with previous studies [10, 22]. The lack of proteolytic enzymes might limit the invasion of brucella in BS. Further researches demonstrated that osteoblastic activity is induced in BS, partly explaining the less prominent bone and disc destruction than in TS. It was significantly higher of vertebral hyperplasia with BS (96.15%) than TS (29.63%). There was distinctly more vertebral hyperplasia (Fig. 5a, b) in our result when compared to previous studies [11, 17]. The bone erosion of the endplate in BS was accompanied by new bone formation at the early stage [23]. As a result, the corresponding signs in anterior osteophyte and sclerosis were observed on MR images [21].

The abscess, around the vertebra, is a common feature in BS and TS. Our study found that the paravertebral abscess was significantly higher with BS (65.38%) when compared to TS (22.22%), but the psoas abscess was markedly lower with BS (0.00%) when compared to TS (66.67%). There is a significant difference between BS and TS in terms of abscess spread. The abscess beyond the range of vertebral lesions was significantly higher in TS (94.44%) when compared to BS (5.88%). Small abscesses were frequently found by Tali et al. [21]. because the abscess in BS is relatively limited, it is generally difficult to spread. About 34.62% in BS showed epidural abscesses, which followed a previous study [24].

Previous studies [2, 12, 25, 26] showed the diagnosis and differential diagnosis in spondylitis patients on MRI was qualitative rather than quantitative. MR T2 mapping can be used to detect the early changes in physiology and morphology by water content changes in the tissues and indirectly reflect the small changes of water molecules of the tissues in the spatial information of human tissue structure and pathological and physiological conditions [27]. Spondylitis is often caused by brucella or tubercular bacteria, resulting in inflammatory vertebral edema as well as abscesses, and the paravertebral abscess can result in the increased random Brownian motion of water protons, which is reflected by increased T2 values. To the best of our knowledge, MR T2 mapping has been used to evaluate vertebal injury in spinal tuberculosis [9]. However, there was no similar research on the application of T2 mapping between BS and TS. In our work, the results showed that T2 value of the LV with BS was markedly higher than that in the LV with TS (*p*  <  0.05) and that in the UAV with BS (*p*  <  0.05). The T2 value of the LV was high in BS and TS. The reason may be the bacteria entering the vertebra through the blood to undergo a complex pathological inflammatory process (seep, hyperplasia, and necrosis). With the inflammatory pathological lesions developing, the extracellular water content increases, and the injured locations present congestion and edema of different degrees. As the vertebas have abnormal pathological changes, MR T2 value was increased by T2 relaxation time extended. In our work, the T2 value of the LV in BS was higher than that in TS. So, we had a preliminary result that T2 mapping may quantitatively differentiate BS from TS.

## Limitations

Due to the small sample size included in this study, MR T2 mapping sequence scanning needs to be further studied by expanding the sample size in the diagnosis and differential diagnosis with BS and TS. Another limitation was that we did not determine the stage of disease between BS and TS in this series. Therefore, the T2 value may be potentially inaccurate.

## Conclusion

The qualitative and quantitative evaluation may differentiate BS from TS. The conventional MR imaging helps to distinguish BS from TS by several distinctive features. MR T2 mapping has the additional potential to provide quantitative information between BS and TS.

## Abbreviations

BS: Brucella spondylitis
TS: Tuberculous spondylitis
MRI: Magnetic resonance imaging
LV: Lesion vertebrae
UAV: Unaffected adjacent vertebra
STIR: Short-tau inversion recovery
ROI: Region of interest
